# Cell-Free Expression and *In Situ* Immobilization of Parasite Proteins from *Clonorchis sinensis* for Rapid Identification of Antigenic Candidates

**DOI:** 10.1371/journal.pone.0143597

**Published:** 2015-11-24

**Authors:** Christy Catherine, Seung-Won Lee, Jung Won Ju, Ho-Cheol Kim, Hyun-Il Shin, Yu Jung Kim, Dong-Myung Kim

**Affiliations:** 1 Department of Fine Chemical Engineering and Applied Chemistry, Chungnam National University, Daejeon 305–764, Korea; 2 Division of Malaria and Parasitic Diseases, National Institute of Health, Osong 361–951, Korea; University of Saskatchewan, CANADA

## Abstract

Progress towards genetic sequencing of human parasites has provided the groundwork for a post-genomic approach to develop novel antigens for the diagnosis and treatment of parasite infections. To fully utilize the genomic data, however, high-throughput methodologies are required for functional analysis of the proteins encoded in the genomic sequences. In this study, we investigated cell-free expression and *in situ* immobilization of parasite proteins as a novel platform for the discovery of antigenic proteins. PCR-amplified parasite DNA was immobilized on microbeads that were also functionalized to capture synthesized proteins. When the microbeads were incubated in a reaction mixture for cell-free synthesis, proteins expressed from the microbead-immobilized DNA were instantly immobilized on the same microbeads, providing a physical linkage between the genetic information and encoded proteins. This approach of *in situ* expression and isolation enables streamlined recovery and analysis of cell-free synthesized proteins and also allows facile identification of the genes coding antigenic proteins through direct PCR of the microbead-bound DNA.

## Introduction

Despite the advances in the diagnosis and treatment of many types of human diseases, there are a number of parasite-related diseases that still remain substantial threats to public health. Unfortunately, there is a lack of reliable diagnostic and prognostic markers for many parasitic diseases, and post-symptomatic treatment relies on the use of only a few compounds. Furthermore, complex life cycles and highly adaptable gene expression mechanisms within parasites create the challenge of drug resistance. For instance, malaria parasites have developed resistance even to the promising compound artemisinin [1]. There is also a growing concern that global climate change could accelerate the prevalence of parasitic diseases [2]. Therefore, there is a pressing need for new diagnostic and therapeutic tools to reinforce the present arsenal for combating major parasitic diseases.

Advances in vaccine development and drug discovery can be facilitated by genome-scale production of purified recombinant proteins from parasites, which has become possible by recent achievements of sequencing projects [3,4]. However, the expression of parasite proteins on large scale is a challenging task because traditional cell-based methods are slow and often inefficient for the expression of foreign proteins that are soluble. As an alternative approach to produce recombinant proteins, cell-free expression systems offer numerous advantages over cell-based methods [5, 6]. Because the approach of cell-free synthesis does not require many of the time- and labor-intensive steps required of conventional cell-based protein expression, parasite proteins can be readily produced and analyzed from various types of DNA templates [7–11]. Tsuboi et al. used a wheat germ cell-free protein synthesis system for the parallel expression of PCR-amplified genes of *P*. *falciparum* [12]. The wheat germ expression system has also been used for antigen discovery in *P*. *vivax* [13,14]. In addition, using an *E*. *coli* cell-free system, Doolan and co-workers reported the expression of *P*. *falciparum* proteins subsequently coupled to solid arrays and analyzed with immune sera, which led to the discovery of putative new antigens [15].

In addition to the benefits of speed and convenience in protein production, as demonstrated in previous work, the open nature of cell-free synthesis allows for simple modifications of the system with various biological and non-biological components to mix and match steps and conditions. For example, the steps of protein expression and purification can be integrated simply by placing affinity beads in the reaction mixture during the protein synthesis [16]. Cell-free protein synthesis can also be controlled by conjugating template DNA on magnetic particles and moving them in and out of the reaction mixture to turn synthesis on or off, respectively [17].

In this study, we exploited the open nature of cell-free synthesis for the rapid identification of structural antigenicity of parasite proteins from the genes of *Clonorchis sinensis* (*C*. *sinensis*), which is the parasite responsible for clonorchiasis disease. Although not as deadly as malaria, clonorchiasis is a disease that substantially burdens human populations in the regions including Korea, Taiwan and Southern China [18]. For the preparation of template DNA for the synthesis of parasite proteins, parasite genes were PCR amplified with biotinylated primers and conjugated on microbeads that had been dual-functionalized with streptavidin and Ni-NTA. Upon the incubation of the microbeads, target proteins were expressed from the microbead-conjugated DNA and instantly immobilized onto the same microbeads, thereby becoming physically linked to the encoding genes. Microbead-captured parasite proteins were recovered from the reaction mixture and examined for the antigenicity against patient sera. The DNA coding the potentially antigenic proteins was readily identified by PCR of the microbeads, showing positive antigenicity against the patient sera. Compared to conventional recombinant DNA methods, this approach allows for rapid analysis of the structural antigenicity of unknown proteins from parasite genes with minimal processing time. It thus can be used for large-scale screening of antigenic candidates from sequenced parasite genomes.

## Materials and Methods

Human antisera against *C*. *sinensis* were prepared from patients with clonorchiasis. This study involving the use of the patient sera was approved by the Research Ethics Committee of Korea National Institute of Health (approval no. 2013-06EXP-04-R). Authors confirm that written consents were obtained from the donors for the use of serum samples for research purpose.

### Materials

ATP, GTP, UTP, CTP, creatine phosphate, and creatine kinase were purchased from Roche Applied Science (Indianapolis, IN). Streptavidin-coated microbeads were purchased from Thermo Scientific (Rockford, IL). L-[U-^14^C] Leucine was from Perkin Elmer (Waltham, MA). FITC-conjugated murine anti c-myc antibody was purchased from Santa Cruz Biotechnology (Dallas, TX). Biotin X-NTA was from Anaspec (Fremont, CA). All other chemical reagents were obtained from Sigma-Aldrich (St. Louis, MO) and used without further purification. The S12 extract prepared from the *Escherichia coli* strain BL21 Star (DE3) (Invitrogen, Carlsbad, CA) was used as a source of protein synthesis machinery, as described previously [19]. Human antisera against *C*. *sinensis* were prepared from patients with clonorchiasis. This study involving the use of the patient sera was approved by the Research Ethics Committee of Korea National Institute of Health (approval no. 2013-06EXP-04-R).

### Preparation of templates for cell-free synthesis of parasite proteins

14 *C*. *sinensis* genes, cloned in frame with C-terminal histidine tag in pET and pRSET vectors (Table 1) were PCR amplified using the primers against the T7 promoter and the T7 termination sequences. Amino acid and nucleotide sequences of the examined proteins are given in S1 and S2 Tables, respectively. For the preparation of bead-immobilized DNA templates, PCR reactions were conducted with 5’ and/or 3’-biotinylated primers. The PCR products (70 μg/mL) were mixed with the same volume of streptavidin-coated microbeads and kept at room temperature for 1 h with constant agitation. After being washed in PBS buffer, the DNA-conjugated microbeads were incubated with 0.2 mg/mL of biotin X-NTA for the conjugation of unoccupied binding sites of streptavidin with NTA. To charge the NTA group with nickel, the DNA-NTA-conjugated microbeads were incubated in 25 mM NiCl_2_ for 30 min at room temperature. The resulting microbeads were washed and stored in PBS buffer prior to use.

**Table 1:** *C*. *sinensis* genes used in this study.

ID No.	Name	Cloned vector	Expected protein size (kDa)
C1	Legumain	pET28_A	47.5
C2	Tegumental protein 20.8kDa	pRSET_B	20.3
C3	Tegumental protein 20.6 kDa	pRSET_B	19.5
C4	Tegumental protein 31.8kDa	pRSET_B	30.3
C5	Tegumental protein 21.6kDa	pRSET_A	20.8
C6	Tegumental protein 22.3kDa	pRSET_A	21.5
C7	Venome allergen-like protein 1	pRSET_A	24.9
C8	Venome allergen-like protein 3	pRSET_A	43.6
C9	14-3-3 protein	pRSET_A	27.3
C10	Propionyl-CoA carboxylase	pRSET_A	58.9
C11	Prohibitin	pRSET_A	30.6
C12	Triosephosphate isomerase (TIM)	pRSET_A	27.8
C13	Hepatocellular carcinoma-associated antigen 59	pRSET_B	33.9
C14	Ribonucleoprotein antigen	pRSET_A	33.4

### Cell-free protein synthesis of parasite antigen candidate proteins

The standard reaction mixture (150 μL) for the cell-free synthesis of the parasite proteins consists of the following components: 57 mM HEPES–KOH (pH 7.5); 1.2 mM ATP; 0.85 mM each of GTP, UTP, and CTP; 80 mM ammonium acetate; 34 μg/mL 1-5-formyl-5,6,7,8-tetrahydrofolic acid (folinic acid); 1.0 mM each of the 20 amino acids; 2% PEG (8000); 3.2 U/mL of creatine kinase; 67 mM creatine phosphate; 0.01 mM L-[U-^14^C] leucine (11.1 GBq/mmol); 27% (v/v) of the S12 extract; and 26.7 μg/mL of the PCR-amplified template genes. The reaction mixture was incubated at 30°C for 3 h. For the cell-free synthesis and co-immobilization experiments, 30 μL of DNA-conjugated microbeads, prepared as described above, was used instead of the PCR products. The amount of synthesized protein was determined by measuring the TCA-insoluble radioactivities using a Tri-Carb 2810TR liquid scintillation counter (PerkinElmer, Waltham, MA), as described previously [19]. Cell-free synthesized proteins were also analyzed on 16% Tricine gels [20].

### Determination of the antigenicity of the cell-free synthesized proteins

After the cell-free synthesis reactions, the microbeads were recovered by a brief centrifugation and washed 3 times in excess PBS buffer. For the primary confirmation of *in situ* immobilization of the cell-free synthesized proteins, the recovered microbeads were treated with FITC-conjugated anti-myc antibody for 1 h. After being washed in excess PBS buffer, fluorescence from the microbeads was monitored under a fluorescence microscope. To determine the antigenicity of the bead-immobilized proteins, the microbeads were incubated with patient sera diluted in TBST buffer for 1 h at room temperature. After being washed in PBS buffer 3 times, the microbeads were treated with HRP-conjugated goat anti-human IgG. The PBS buffer-washed microbeads were then supplied with a luminol/H_2_O_2_ solution to generate chemiluminescence signals. The chemiluminescence signals were imaged and quantified by LAS-3000 image analyzer (Fuji Film, Tokyo, Japan).

## Results and Discussion

### Cell-free synthesis and Western blot analysis of parasite proteins

**Fig 1:**
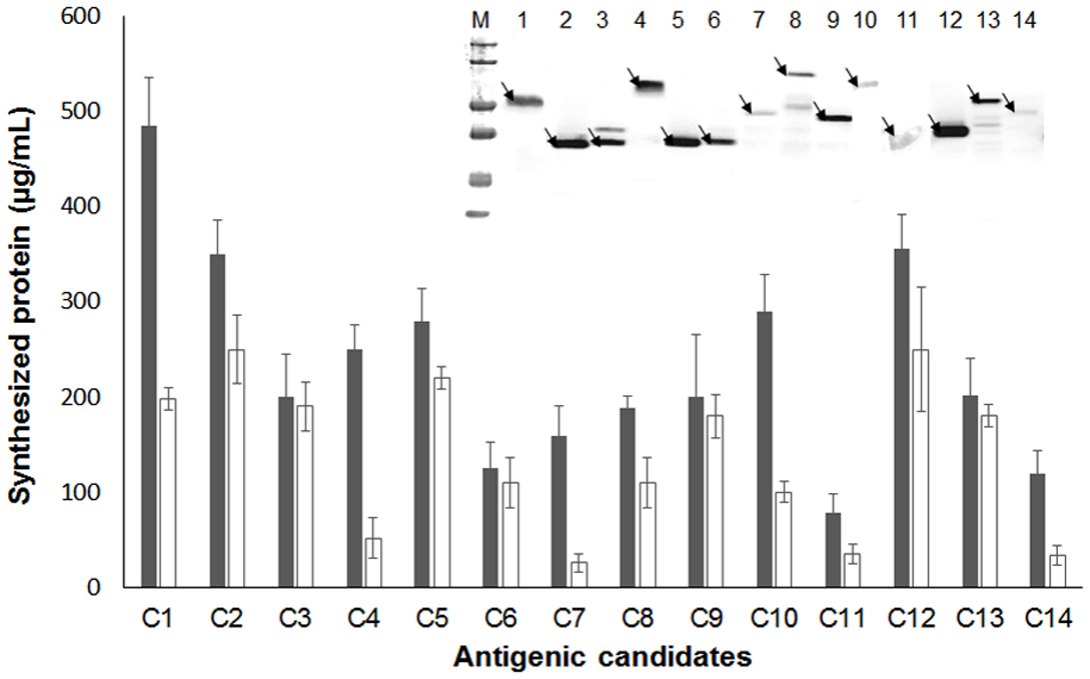
Cell-free expression of *C*. *sinensis* proteins. Target genes were PCR amplified from the cloned vectors and used as the templates for the cell-free protein synthesis. The synthesized proteins were quantified by measuring TCA-insoluble radioactivity, as described in the Materials and Methods. Each of the bars in the graph shows average measurements based on three replicates of the experiment. Filled bars represent the total amounts of cell-free synthesized proteins. Blank bars represent the amount of soluble fractions of the synthesized proteins that were determined by measuring the radioactivity in the supernatant of the samples after centrifugation at 4,000 RCR for 10 min. The error bars represent the standard deviation of the measurements. The reaction samples were also analyzed by Western blot using anti-6xhis antibody as shown in the embedded image in the graph.

**Fig 2:**
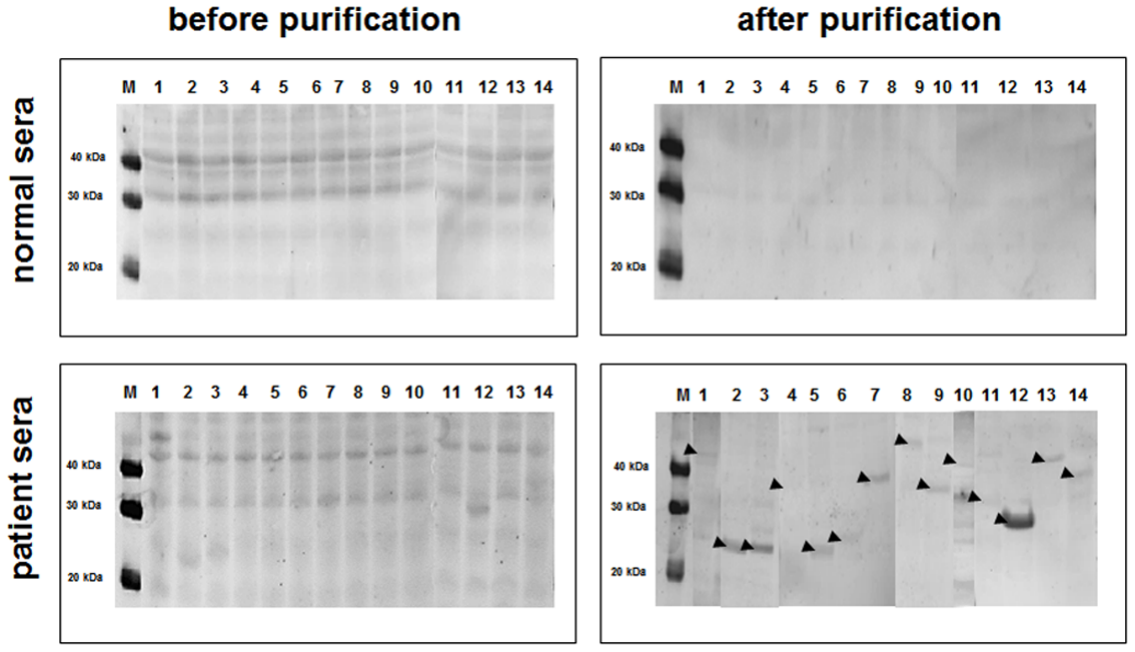
Western blot analysis of cell-free synthesized *C*. *sinensis* proteins. Cell-free synthesized proteins were analyzed by Western blot before and after purification. Blotted proteins were incubated with mixed sera of Clonorchiasis patients or normal sera followed by detection with HRP-conjugated murine anti-human IgG.

When incubated under standard reaction conditions, all of the PCR-amplified target parasite genes were successfully expressed with varying amounts of total and soluble fractions (Fig 1). However, while direct Western blot analysis of the synthesis reaction mixture is desirable for seamless detection of the cell-free synthesized proteins, the proteins were not detected as distinctive bands without purification due to the masking of the target bands with heavy background signals from the S12 extract (Fig 2).

### *In situ* immobilization of cell-free synthesized proteins

**Fig 3:**
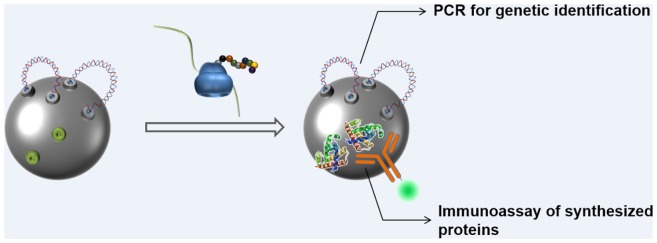
Schematic representation of the concept for cell-free synthesis, *in situ* immobilization, and immunoassay of proteins. Proteins synthesized from microbead-tethered DNA are instantly immobilized on the same surface of the microbeads during the cell-free protein synthesis reactions. Microbeads are recovered from the reaction mixture and analyzed for the antigenicity of the bound proteins and DNA.

The open nature of cell-free protein synthesis allows for the direct addition of various materials into the reaction mixture. This unique feature of cell-free protein synthesis was previously used for coupling protein synthesis with *in situ* isolation of expressed proteins and peptides [16]. By conducting cell-free protein synthesis reactions in the presence of affinity beads, synthesized proteins could be instantly recovered from the reaction mixture. In our study, protein synthesis was conducted in the presence of microbeads conjugated with template DNA to screen the antigenicity from parasite genes. Because the microbeads were functionalized with Ni-NTA groups as well as streptavidin, it was expected that the expressed proteins with histidine tags would be captured on the same microbeads, which were physically linked to the template DNA on the surface of the microbeads. Fig 3 summarizes this approach. In a preliminary experiment using sfGFP as a model protein, the open reading frame (ORF) of sfGFP cloned between the T7 promoter and T7 terminator was amplified with a biotinylated forward primer and an unlabeled reverse primer or biotinylated forward and reverse primers. When the PCR-amplified DNA was conjugated on the microbeads and incubated in the reaction mixture for cell-free synthesis, as expected, sfGFP fluorescence was observed from the recovered microbeads, indicating successful *in situ* immobilization of the cell-free synthesized proteins.

**Fig 4:**
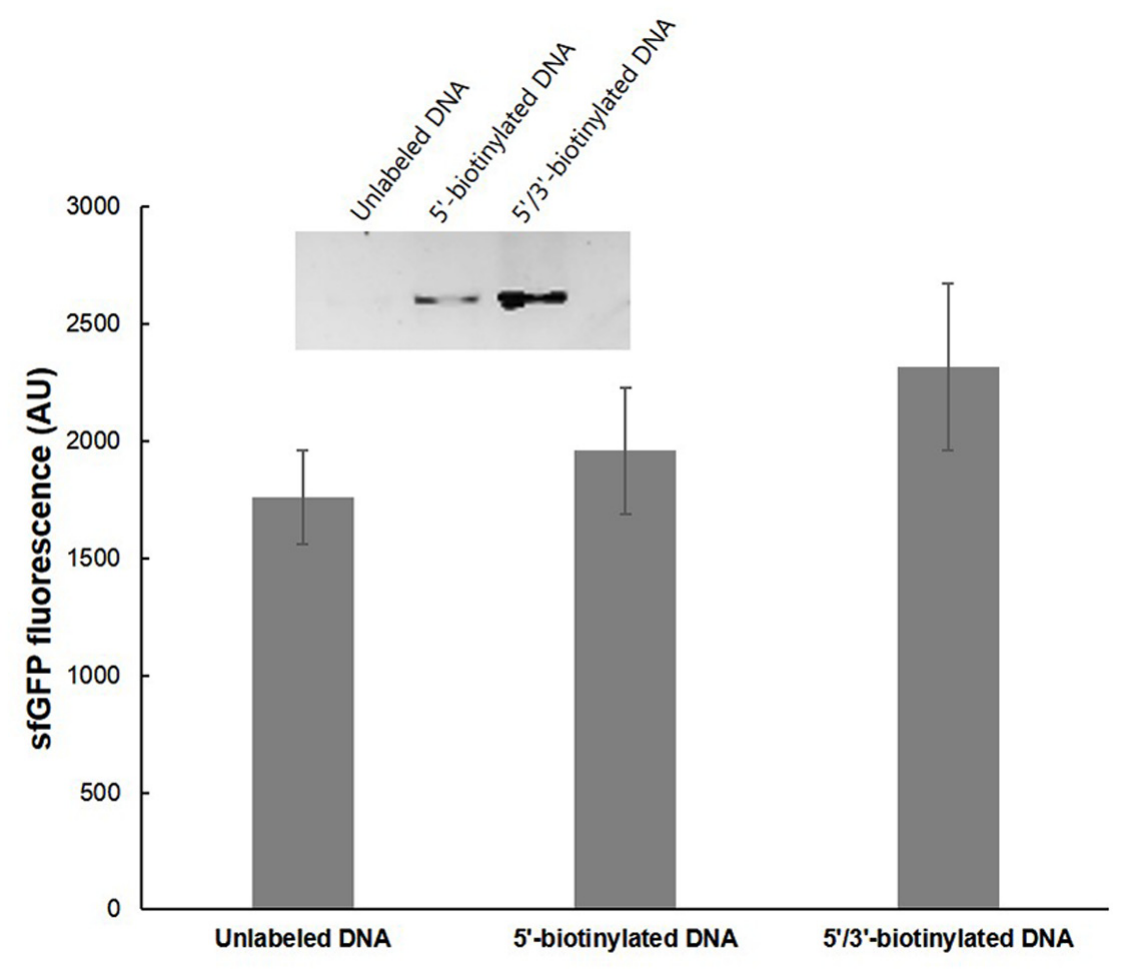
Effect of DNA immobilization on the re-amplification of template DNA after cell-free protein synthesis. 5’- or 5’/3’-biotinylated sfGFP genes were conjugated on microbeads and incubated in the reaction mixture for cell-free protein synthesis. After a 3-h incubation, the sfGFP fluorescence from the reaction mixture was measured using a fluorospectrometer at 485 nm excitation and 535 nm emission wavelengths. The reaction samples were also analyzed by SDS-PAGE as shown in the embedded image in the graph. The same reaction mixtures were then PCR amplified to recover the template DNAs.

In general, PCR products are rapidly degraded in *E*. *coli* extract by exonuclease activity. Although the short lifetime of the template does not lead to a substantial reduction in protein productivity due to high mRNA stability in the system, it would make it difficult to re-amplify DNA after cell-free synthesis reactions. However, conjugation onto a solid surface can restrict the access of nucleases to the termini of the PCR products, thereby keeping the DNA intact for longer periods of time. Based on this presumption, it is reasonable to expect higher stability of dual-labeled DNA than free or single-labeled DNA. The results of PCR amplification of the microbead-conjugated DNA after a 3-h incubation of the reaction mixture strongly supported this presumption. While the free template could not be re-amplified after the incubation period, the same template DNA tethered on a microbead was able to be re-amplified after the protein synthesis reaction. In particular, the double-labeled DNA gave clearer amplification products than the single-labeled DNA (Fig 4). Therefore, for the maximum synthesis of target proteins and facile identification of the encoding genes by PCR after the synthesis reactions, we used biotinylated forward and reverse primers in the subsequent experiments for the expression of antigen candidates.

### Cell-free synthesis and *in situ* immobilization of antigenic proteins

**Fig 5:**
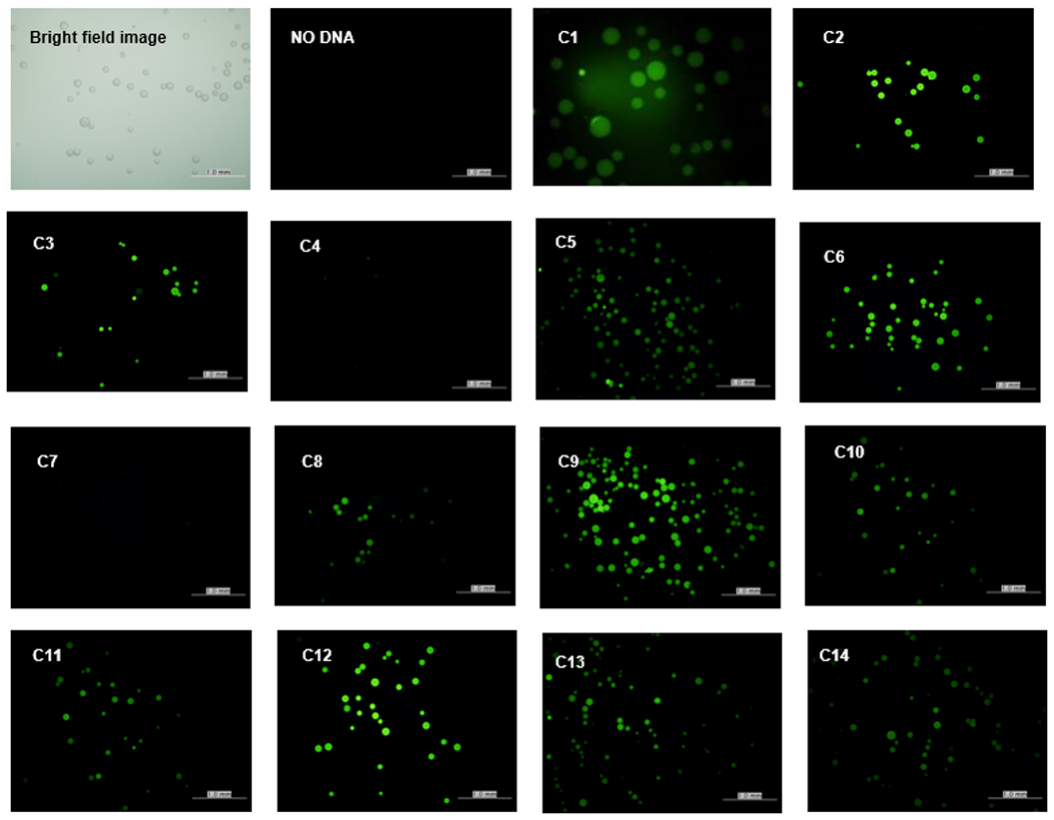
Cell-free synthesis and *in situ* immobilization of *C*. *sinensis* proteins. Fourteen genes of *C*. *sinensis* were PCR amplified with biotinylated forward and reverse primers and conjugated on streptavidin-coated microbeads. Vacant sites of the microbeads were then conjugated with biotin-NTA. After charging the NTA group with Ni^2+^, the microbeads were incubated in the reaction mixture for cell-free protein synthesis. The proteins captured on the microbeads were detected with FITC-conjugated anti-myc tag antibody, as described in the Materials and Methods

In the subsequent experiments to investigate the feasibility of cell-free synthesis and *in situ* immobilization of antigen candidates, a myc-tag sequence was added to the ends of the target genes during the first PCR step. The microbeads conjugated with template DNA were incubated in the reaction mixture for the cell-free protein synthesis, washed in PBS buffer, and analyzed after the addition of the FITC-conjugated anti-myc antibody. As expected, most of the cell-free incubated microbeads were found to be fluorescent, indicating the presence of synthesized proteins on the surface of the microbeads. The microbeads exhibited varying intensities of FITC fluorescence depending on the proteins encoded on template DNA (Fig 5). The lack of significant fluorescence in two samples (C4 and C7) was thought to be due to the low solubility of the expressed proteins or limited accessibility of the myc-tag to the antibody. In other cases, relative intensity of the microbeads correlated with the levels of soluble proteins determined using a liquid scintillation counter.

### Examination of the antigenicity of the microbead-immobilized parasite proteins

**Fig 6:**
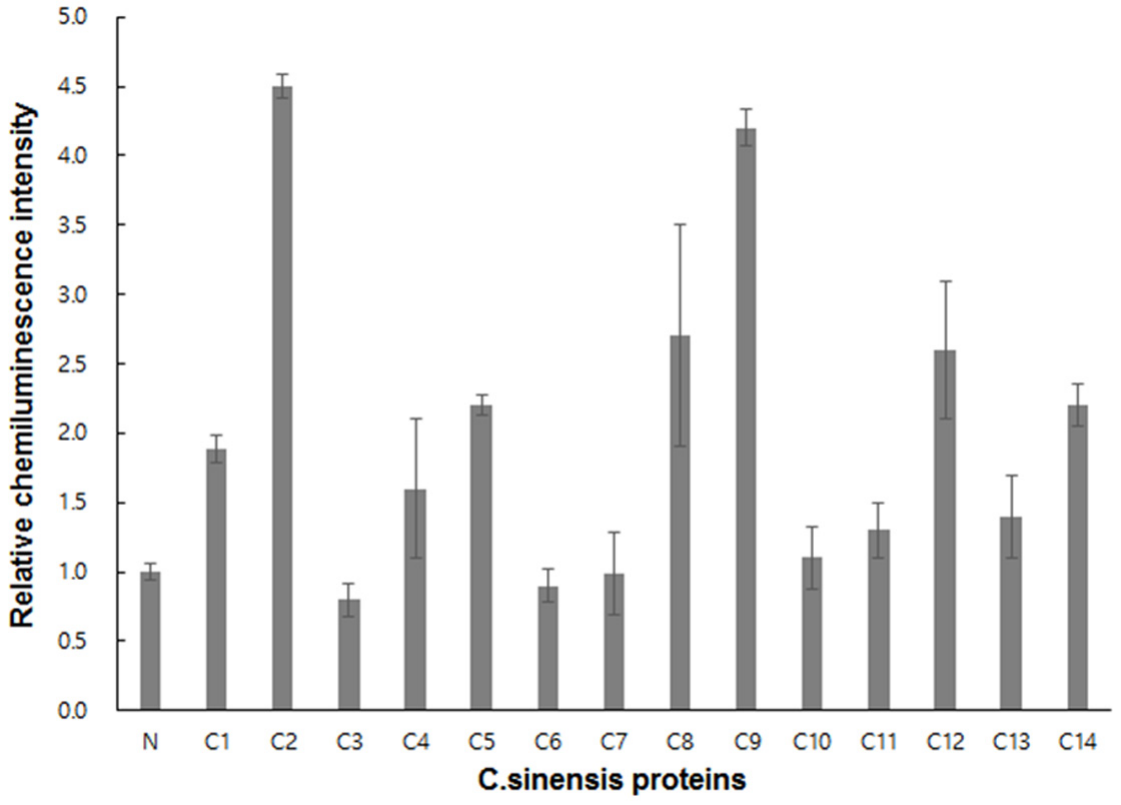
Cell-free synthesis and screening of in situ immobilized *C*. *sinensis* proteins. The microbeads conjugated with *C*. *sinensis* genes and Ni-NTA were incubated in the reaction mixture for cell-free protein synthesis in each well of a 96-well plate. After the synthesis reaction, the antigenicity of the bead-captured proteins was determined by sequential treatments with patient sera and HRP-conjugated anti-human IgG antibody. Chemiluminescence upon the addition of luminol to each well was imaged and quantified. Data represent the results from three replicates of the experiment.

After confirming the possibility of streamlining the expression and screening of antigenicity, as described above, we next proceeded to examine the antigenicity of cell-free-synthesized and *in situ* bead-immobilized *C*. *sinensis* proteins using patient sera. The reactivity of the patient sera to the proteins immobilized on the microbeads was determined using murine anti-human IgG conjugated with HRP, as described in the Materials and Methods. As shown in Fig 6, this resulted in 6 out of 14 *C*. *sinensis* proteins giving chemiluminescence signals more than twice the intensity of the control assays where uninfected sera was used. It is interesting that some of the examined proteins failed to give signals higher than the control assays because all of them were found to be antigenic to the same patient sera in the Western blot assay of the purified proteins (Fig 2). Assuming that the proteins immobilized on the microbeads are in folded states, the results from the assay of the bead-immobilized proteins might represent more realistic antigenicity because the results of the Western blot analysis of the reduced and denatured proteins include the reactivity against sequential antigenic determinants that might not be present in the folded state of the proteins [21].

## Conclusions

Many different versions of cell-free protein synthesis systems have been developed and improved to provide high-quality proteins in a high-throughput manner. Screening of vaccine candidates is one of the areas where cell-free protein synthesis can be implemented as a pivotal tool for converting massive sequence information into functional proteins. While the direct detection of the antigenicity of cell-free synthesized parasite proteins was hampered by the lack of sensitivity and specificity during the assays, we have developed an approach that combines the unique advantages of cell-free protein synthesis and the accuracy of ELISA assay. Our results show that parasite proteins can be efficiently expressed from microbead-conjugated DNA and displayed on the same microbeads to enable rapid determination of their conformational antigenicity without the interruption of purification steps. Although the issue of soluble expression remains to be addressed for certain proteins, we expect that the presented methodology can be provide an option for rapid screening of antigenic proteins from genetic libraries of various sources.

## Supporting Information

S1 Table Amino acid sequences of *C*.*sinensis* proteins. (DOCX)

S2 Table Nucleotide sequences of *C*.*sinensis* proteins. (DOCX)
