# Supplementary material for: Cell-Free Expression and In Situ Immobilization of Parasite Proteins from Clonorchis sinensis for Rapid Identification of Antigenic Candidates
Source: PLoS One. 2015 Nov 24;10(11):e0143597. doi: 10.1371/journal.pone.0143597 (PMC4657965; doi:10.1371/journal.pone.0143597)
Supplement: S1 Table — (DOCX) [file pone.0143597.s001.docx]

**Supporting Information**

Cell-free expression and *in situ* immobilization of parasite proteins from *Clonorchis sinensis* for rapid identification of antigenic candidates

Christy Catherine^1,†^ Seung-Won Lee^1, †^, Jung Won Ju^2^_,_ Ho-Cheol Kim^1^, Hyun-Il Shin^2^, Yu Jung Kim^2^ and Dong-Myung Kim^1,*^

^1^Department of Fine Chemical Engineering and Applied Chemistry, Chungnam National University, Daejeon 305-764, Korea

^2^Division of Malaria and Parasitic Diseases, National Institute of Health, Osong 361-951, Korea

^†^These authors equally contributed to this work

^*^Corresponding author

E-mail: [dmkim@cnu.ac.kr](mailto:dmkim@cnu.ac.kr)

S1 Table. Amino acid sequences of *C.sinensis* proteins

| ID No. | Amino acid sequences |
| --- | --- |
| C1 | WLGSVCVGSRLLHSDPTKNWVVLVAGSNGWGNYRHQADVFHAYQILRHNNISAEQIITFAYDDIANNSENPFMGKVFNDYYHIDVYEGVIIDYRGEDVTPQNFLRVLRGDKELEAAGKKVLKSGPEDHVFIYFSDHGGDGIISFPEDELSATDLNKTLGYMYKNGKYKKLVLYVEACESGSMFEGILPSNIGIYVTTAANNQEASWATFCHDEVIDTCLADEYSYNWLTDSEEHDLTHRTLDQQFKSVKRRTKRSHVSRFGEMDVGRLPVGDFQGHSEQSMLLDSATMTQVLHSRPSRWAHLTTISRRLVHAESVEEHELAARKLYRTLQLGHIVKQTFDDIVMDVTTFHQPTIHELSKSEELQCYEAVFKQFRKRCFTIRQVPEVAQYAGYLRKLCKKGYETKILIQSVHKV |
| C2 | MDSFINIFVSIDKDGTNVISYPELEQYVAENNLDPSMVEKWKQLFDPDNTGSITLETFCSKLGLKPAEIIDFREQKGLHAAPPSLPPEIIVISANMSLEDQIKIARETIPIAPGAQTSEELGRLTENLKSFADKTFGGCWQVMVVDGSYWITQTFVPNMSFQFELYNRAYLFWQTSEDEVALAQ* |
| C3 | MEPFLEAFFSIDTDHTERITIRELQDYVRRNNIDPSMIKRWQVLFDADDSGVITLDEFCKTLGIRPSEARAYNANMVRASRGPSLPREVDVITATLPLDQQVDIVNEVMRLTRNEPFDENLVSKQLKQFLDRQYGRMWHVVITKGSSWCSFSYEPKTSLFFQLRKYTYLVWKTPS |
| C4 | MDAFIEAFYAIDVDRSETITLDELRNYMEKNNMDPAFIERWQEIFDPEHTGSITLNSFCEVLGLELNNIRGQFDAAESVKQSASKQNDDDEDRERSPPPQNGKLKQDDKYDERARSNPSESADEHNIWLEQKESSPLAFVNGDQTKQTSDESFDNMRVDETAPFSALDKGSDDSQKKIMENGLGYGNGYEEISVDIGQELKTAIVHYAIEGLGLHQEDRDLVKWLKQRMDKEHGRLWHCTIVRGQYFSFYSYQPGHSFCFKIGPRIFIIFKTPYY |
| C5 | MGEQGSDMEKMIEMFLGMDKNDDGFVDLSELRTACQEKKLDMKQVNGWLSRYDTNKDGKISLDEFCDGLGLGKQEMIVEKEERDISNTKVCPTIAHEIKPLDTTMSIAKQAHITDKFIELAKEVSSDPHKMNQVAAKMKRFLDEQYGRVWQVIVLAGSYWINYSHAPFLSMQFQYGPYICIVWRTTIN |
| C6 | MSYTPSQLERLILKFLELDTNRDEVVDRRELKYAWLNDGITEDEVSHWLDKYDLNGDGNITLDEFCHALGLKCEEMRIERYERQREREGFAKVLNPDVSIIASTMSLDKQVDITNKFVELLKETSGRPEDLNEVAKNLKDYLDKQYGRVWQTVLVAGSYWMKFSHEPFMSLQFKCGPHICLVWRTPCIERDSFN |
| C7 | MLDTELNTQAIALHNQFREKHGSPPLVYDAKLAQTAQNWAEQLAQTKCMRHSDMETYGEN  LAYKGAWENATITGEEATKSWYAQGDYHDFNESFTYETSYFSQLIWKGSKNVGFGRAVSEDGEAAYIVAHYFPKGNIRSVFSNNVPKLCSAPSANTTGTPVSTPNMRYTKLETKKELKEREKAEKKARERAEKERKEREKQLKKEQKEREKQAKKDKLKSKSLSGI |
| C8 | MPNMDDFIEECLREHNAKRELHGAPALKHSRALDKTAQDWAEALISEPSIKNSPLSSRGEVGESISMRTSSASHVDIQGNEVVNQWYADIKNYNFAEGKGPAGNFTQLVWKATREVGFGKARSSGKCIVVAHYRPPGNVRGHYAENVGTPTGEQAASVASATDTGNLDPNAKRTVVTEEVTSPEGKRYTVHREVIETTEPDGHVRRCVNETFQDSPDQATAGGKHGASSEAAHGENFADAVTRAHNVYRKRHGVADLQLDPEISHMAQDWAEQLVNRAHLSNSGFTYQGVRLGENVLCRWSNTAATVSAQDVVDHWYQESSKYKFNSEPKSIQGIGGFTQMVWNGSQRIGVGIASQAKKDFYNQPSQSKVIVVCFYYPPGNVTGQFRANVKQGMN |
| C9 | MVSDESWVVRENISDPEALLHIAKLAEHAERFTDMAAAMKKFTELKKPLSNDERNLFSVAYKNVVGARRSAWRVITSIKNKDSEDEKSPTNELRRKIENELEQVCKEVLNILEKNSLPSETTDDGLVFYHKMKGDYYRYLAEVQTGDKRNESVQKSHQAYEEATEKAKQSLSETHPIRLGLALNYSVFYYEIENNPDKACELAKSAFDNAISRLDQIKDESYKDSTLIMQLLRDNLTLWTSERETDQ* |
| C10 | MLHFARNSLRLLSVARTPNLRFISGAAHTLEVRHKIEETREKIVLGGGQKRIDAQHKRGKLTARERIDLLADPDTFVEYDAFMEHDCHDFDMQRQRVTGDSVVTGHCQVNGKTVYLFSQDFTVFGGSLSLVHARKICKVLDQAMLVGAPVIGLNDSGGARIQEGVASLAGYADIFQRNVNASGVIPQISLIMGPCAGGAVYSPALTDFIFMVQDTSYMFITGPDVVKSVTNEDVTQDELGGAKTHCSVSGVAHRAYQNDVEAILSVRDFLTYLPSSNRQKCAPLRECHDPVDRLVPTLNTIVPLEPTSAYDMYEVIYAIVDEREFFEIMPSYAKNLIVGFARLGGRTVGVVANQPRVSAGCLDINCSVKGARFVRFCDAFNIPLITFVDVPGFLPGTSQEYGGIIRHGAKLLFAFAEATVPKLTVTTRKSYGGAYCVMSSKHLRGDINYAWPSAEVAVMGAKGAVQIIFRGKEDQAQAEEDYIRTFANPFPAAVRGYMDDILDPPLTRKRLCHDLEMLQTKSLTNPWKKHANMPL* |
| C11 | MAQLNAMFGRLVKLGVGIVAAGSILPMVLYNVDGGHRAVIFDRFKGVHPEVVGEGTHFIIPWVQKPIIFDIRSKPRNIPVMTGSKDLQTVNITLRILFRPESSLLPKIYQNLGFDYEERVLPSITTEVLKGVVAQFDASELITQRELVSQRVNDDLTERASSFGILLDDIALTQISFGREFSEAVEAKQVAQQEAERARYLVEKAEQQKLAAVITAGGDSEAATLLAKAFGSSGEGLIELRRIEAAEDIAYQLSKNRNVTYLPEGQHTLLNLPSVQT* |
| C12 | MPTDRKFFVGGNWKMNGSKKENDKLIEMLTHAKIDPNTEVLVAPPALYLPSVREKLDKRFHVAAQNCYKVPSGAFTGEVSPAMLKDVGCDWVILGHSERRHILLETDQLVGEKTNHAISAGVNVIACIGEKLEEREAGKTEEVCFRQMEAIRKNLSSADMWNHIVIAYEPVWAIGTGKTATEQQAQEVHLAVRRWMEEKVSPAVAKSIRIIYGGSVTAANCRTLAKQPDVDGFLVGGASLKPDFIEICNANA |
| C13 | MDSAKKRCLRTRRPSDSSESEEEKTNDSNEIVQQEDSTHVVEAIRELQKVRKRPPGISLSALSTGKAAPEETIIVSDPFKLKTGGLVEIRKAIRSKKTEEEDDVEARLAKTFATETNKRDEDAEMIKYIEEEIARRKGLRRTPSPESNAGADLLRDVPEYLRPVIGQQKEDMLSNQMLCGIPEVDLGVDAKMRNIEATEEAKQTLLKHRFNRGYGMASDGLAPTNVAVNFVQHSRWNSHNATTTFSSGDYTRDLLSIASKANPHKTDIVHQQTTGLDAERERLGAERSTDSLVLQRFKSHMRGRKRR |
| C14 | MTQFLPPNLLALFAPRDPVPFLPPIEKHAHHRKLPYTGVAQFLGEFEDPSETKPAVRIETREERKERKRREKQEQANYKLEQDLALWNPKKNPSATSNAYNTMFVARMNYDTSEGKLRREAEAFGRVTQIVMVKNRLTGKPRGYAFVEFEHEREMHAAVKGLNGKKIDGMRILTDIERGRTRPDWRPRRLGKGLGKNRQGPSEKPKVSSKHENGREPPSHGRSYGRATGYIREREFDRRKRSRSRSRSRDRDRRRSRSRDRYRKHRSPEGDRSFKRGRDRREDMMRQYGEYGAEIRAEYSGDM |
