# Supplementary material for: Cell-Free Expression and In Situ Immobilization of Parasite Proteins from Clonorchis sinensis for Rapid Identification of Antigenic Candidates
Source: PLoS One. 2015 Nov 24;10(11):e0143597. doi: 10.1371/journal.pone.0143597 (PMC4657965; doi:10.1371/journal.pone.0143597)
Supplement: S2 Table — (DOCX) [file pone.0143597.s002.docx]

**Supporting Information**

Cell-free expression and *in situ* immobilization of parasite proteins from *Clonorchis sinensis* for rapid identification of antigenic candidates

Christy Catherine^1,†^ Seung-Won Lee^1, †^, Jung Won Ju^2^_,_ Ho-Cheol Kim^1^, Hyun-Il Shin^2^, Yu Jung Kim^2^ and Dong-Myung Kim^1,*^

^1^Department of Fine Chemical Engineering and Applied Chemistry, Chungnam National University, Daejeon 305-764, Korea

^2^Division of Malaria and Parasitic Diseases, National Institute of Health, Osong 361-951, Korea

^†^These authors equally contributed to this work

^*^Corresponding author

E-mail: [dmkim@cnu.ac.kr](mailto:dmkim@cnu.ac.kr)

S2 Table. Nucleotide sequences of *C.sinensis* proteins

| ID No. | Nucleotide sequences |
| --- | --- |
| C1 | TGGTTAGGCAGTGTCTGCGTCGGTTCGCGGCTGTTACATAGCGATCCGACCAAAAACTGGGTTGTTCTTGTCGCCGGATCCAACGGTTGGGGTAACTACCGACACCAAGCAGATGTATTTCACGCGTATCAAATCCTGAGGCACAACAATATTTCGGCAGAGCAAATAATTACCTTCGCCTACGATGATATTGCAAACAACTCTGAAAATCCGTTTATGGGCAAAGTGTTCAATGACTATTATCACATAGACGTGTACGAAGGTGTGATAATAGACTATCGTGGAGAAGATGTGACACCACAAAATTTCCTGCGCGTCTTGAGGGGAGATAAAGAACTTGAAGCAGCTGGAAAGAAGGTACTAAAAAGCGGTCCAGAAGATCACGTCTTCATCTACTTTTCCGATCATGGTGGAGACGGAATTATTTCCTTTCCGGAAGATGAGCTCAGTGCCACGGATTTGAACAAAACTTTGGGTTACATGTACAAAAACGGAAAGTACAAAAAACTGGTCCTATACGTGGAAGCATGCGAATCCGGTTCTATGTTCGAGGGTATCCTGCCATCGAATATCGGAATCTATGTGACAACCGCGGCTAACAATCAGGAAGCAAGTTGGGCTACTTTCTGTCACGACGAAGTTATTGATACTTGTCTGGCGGATGAATACTCGTACAACTGGCTCACGGATTCTGAGGAGCACGATCTGACGCATCGTACTTTGGATCAACAGTTCAAATCGGTGAAACGAAGGACCAAACGAAGTCACGTATCCAGATTCGGGGAAATGGATGTGGGCCGTCTTCCAGTCGGAGATTTTCAAGGTCATTCTGAACAATCGATGCTTTTGGATTCGGCTACAATGACTCAGGTACTTCATAGCCGTCCATCACGCTGGGCACATTTGACCACCATCTCCCGACGCCTAGTGCACGCCGAGTCCGTGGAAGAACATGAATTGGCAGCCCGAAAATTATATCGCACACTTCAGCTTGGCCATATCGTCAAACAAACATTCGAC  GACATTGTCATGGATGTAACGACCTTTCATCAGCCAACCATACACGAGTTGTCAAAATCGGAGGAACTCCAGTGCTATGAAGCAGTATTCAAACAATTCAGAAAGCGGTGCTTCACCATTCGACAGGTCCCTGAGGTGGCTCAGTACGCAGGATATCTGCGAAAGCTGTGCAAAAAAGGATACGAAACTAAAATACTCATTCAATCTGTTCATAAAGTCTGTTCCTAG |
| C2 | ATGGATAGTTTTATTAACATATTCGTTTCCATCGACAAAGATGGAACTAATGTAATCTCA  TACCCGGAATTGGAACAGTATGTCGCGGAGAATAATCTGGATCCTTCTATGGTTGAGAAATGGAAACAATTGTTCGATCCAGACAATACGGGTTCAATCACGCTTGAAACTTTCTGCAGC  AAGTTGGGATTGAAACCGGCAGAAATCATTGATTTTCGCGAACAGAAAGGCCTCCATGCA  GCACCTCCAAGCCTTCCTCCAGAAATCATTGTCATCTCAGCTAATATGTCCTTGGAAGATCAGATCAAAATCGCTCGTGAAACGATACCCATTGCACCGGGAGCTCAGACTTCGGAAGAG  CTTGGACGTCTGACGGAAAACCTGAAGTCATTCGCAGATAAAACCTTTGGTGGTTGTTGG  CAAGTAATGGTCGTGGATGGTTCGTACTGGATCACACAGACCTTTGTTCCTAATATGTCCTTCCAGTTCGAACTCTACAACCGAGCCTATTTGTTCTGGCAAACCTCGGAAGATGAAGTG  GCTCTTGCACAATAG |
| C3 | ATGGAGCCATTCTTAGAAGCCTTTTTTAGCATTGACACGGACCACACAGAGAGGATCACT  ATACGGGAGCTGCAAGACTATGTGAGGCGAAATAATATTGATCCGTCAATGATTAAGCGATGGCAAGTTTTATTCGACGCCGACGATTCCGGAGTGATTACACTGGATGAATTTTGCAAGACGCTTGGAATTCGTCCCTCTGAAGCCCGGGCTTACAACGCAAATATGGTTCGAGCTAGT  CGTGGTCCTTCGCTGCCACGCGAGGTTGACGTTATTACTGCAACTCTGCCTTTGGACCAACAGGTTGATATTGTCAATGAGGTGATGCGACTGACGCGCAATGAACCCTTTGATGAGAAT  CTGGTAAGCAAACAACTGAAGCAATTTCTCGACCGTCAATACGGACGAATGTGGCATGTG  GTGATAACAAAAGGATCCAGCTGGTGCAGTTTCTCTTACGAGCCGAAGACCTCACTTTTCTTCCAACTGCGCAAATACACCTACCTTGTGTGGAAGACACCAAGCTGA |
| C4 | ATGGATGCTTTTATCGAGGCATTTTACGCCATTGACGTGGACCGTTCAGAAACGATCACTCTTGATGAGTTGAGGAATTACATGGAGAAGAACAACATGGATCCGGCGTTTATTGAACGC  TGGCAAGAGATATTTGACCCAGAACATACCGGTTCCATCACCTTGAATAGCTTTTGCGAA  GTATTGGGACTGGAATTAAATAACATACGGGGCCAATTTGATGCGGCGGAGTCAGTAAAA  CAATCTGCGTCTAAACAGAATGACGACGACGAAGATCGAGAGCGGTCCCCACCACCACAAATGGTAAACTGAAACAGGATGACAAATATGATGAGCGAGCACGCAGCAACCCATCGGAAAGTGCGGACGAACACAACATATGGTTAGAACAGAAAGAGTCAAGTCCGTTAGCTTTTGTAAATGGGGATCAGACGAAACAAACTTCTGATGAATCATTTGACAACATGCGAGTGGATGAAACCGCTCCGTTTTCTGCATTGGATAAAGGATCAGATGACTCACAGAAAAAAATCATGGAAAATGGTTTGGGATATGGAAACGGATACGAAGAGATATCGGTGGATATCGGGCAGGAGTTGAAAACCGCCATTGTCCATTATGCAATAGAAGGATTAGGCCTGCACCAAGAAGACCGCGACTTGGTCAAGTGGTTGAAACAGAGGATGGATAAAGAACATGGTCGGCTATGGCACTGCACAATCGTCAGAGGCCAGTATTTCTCCTTCTATTCATATCAACCGGGACATTCATTCTGCTTTAAAATTGGACCGAGGATTTTTATTATTTTCAAGACACCATATTATTGA |
| C5 | ATGGGCGAACAAGGATCGGACATGGAAAAAATGATTGAAATGTTTTTGGGAATGGACAAAAACGATGATGGATTCGTGGATTTAAGTGAATTGCGAACCGCCTGTCAGGAGAAGAAGTTGGATATGAAGCAAGTGAATGGTTGGTTGTCAAGATATGATACAAACAAAGACGGAAAAATCAGCCTGGATGAGTTTTGCGATGGTCTTGGTCTTGGAAAGCAAGAAATGATTGTGGAGAAGGAGGAACGCGATATTTCAAACACCAAGGTTTGTCCAACTATCGCTCACGAGATCAAACCACTGGACACAACAATGTCTATAGCCAAACAAGCGCACATTACAGACAAATTTATCGAGTTGGCCAAGGAAGTGTCCAGTGATCCACATAAGATGAACCAAGTGGCTGCGAAGATGAAGCGCTTCTTGGATGAACAGTATGGAAGAGTGTGGCAGGTGATCGTGTTGGCTGGAAGTTACTGGATTAATTACTCTCACGCTCCTTTCCTATCGATGCAATTCCAATATGGACCATACATTTGTATTGTATGGCGTACCACCATCAACTAA |
| C6 | ATGAGCTACACCCCGTCACAGTTGGAAAGGTTGATACTTAAATTTCTTGAATTGGATACT  AACCGTGATGAAGTTGTGGATAGGCGGGAACTCAAATATGCATGGTTAAATGATGGAATAACAGAAGATGAAGTTTCGCATTGGCTGGATAAGTATGACTTGAATGGCGATGGAAATATC  ACTCTGGACGAATTTTGCCACGCACTTGGCTTGAAATGCGAAGAAATGCGTATTGAACGC  TATGAACGTCAACGGGAGCGTGAAGGGTTTGCCAAAGTTTTGAATCCCGACGTGTCGATCATCGCTAGTACAATGTCGTTGGATAAACAAGTGGACATTACAAACAAATTTGTTGAACTA  CTCAAGGAAACATCGGGGCGACCGGAGGATCTGAACGAGGTGGCGAAGAACTTAAAGGACTACCTAGATAAGCAATACGGCCGGGTCTGGCAAACAGTGCTGGTGGCCGGGTCGTACTGGATGAAGTTCTCACACGAGCCATTTATGTCACTACAGTTCAAGTGCGGACCACATATTTGC  CTCGTCTGGCGCACACCATGCATTGAAAGGGATTCATTTAACTGA |
| C7 | ATGTTGGATACTGAGCTGAATACTCAAGCAATTGCTCTTCACAATCAATTCCGTGAGAAA  CACGGAAGCCCACCTCTAGTTTACGATGCAAAATTGGCCCAGACAGCCCAGAATTGGGCAGAACAACTGGCACAGACCAAATGCATGCGGCACAGCGATATGGAAACCTACGGAGAAAACTTGGCCTACAAAGGAGCGTGGGAAAATGCTACTATCACCGGAGAAGAAGCTACAAAGTCTTGGTATGCGCAAGGGGATTATCATGACTTCAATGAAAGTTTCACCTACGAGACGAGCTACTTTAGTCAGCTTATCTGGAAAGGAAGTAAAAATGTTGGATTTGGTCGCGCGGTTTCAGAGGACGGAGAAGCGGCATATATCGTAGCACACTACTTTCCCAAAGGCAACATCCGGAGCGTCTTCTCCAACAATGTTCCAAAACTCTGCTCTGCTCCCTCGGCCAACACAACTGGAACTCCTGTATCCACTCCGAACATGCGGTACACAAAACTGGAAACCAAGAAGGAGCTCAAAGAGCGAGAAAAAGCGGAGAAAAAGGCCCGTGAGCGCGCTGAGAAGGAACGAAAAGAACGTGAAAAACAACTGAAGAAGGAACAGAAAGAGCGGGAAAAGCAGGCGAAAAAAGACAAACTAAAGTCCAAATCGTTGAGTGGAATATAG |
| C8 | ATGCCCAACATGGATGACTTTATCGAAGAATGCCTTCGTGAACATAATGCCAAACGTGAA  TTACACGGTGCTCCAGCTTTGAAACATAGTAGAGCACTTGACAAAACTGCCCAGGATTGGGCGGAAGCTCTGATCTCTGAGCCGTCCATCAAAAACAGCCCCTTATCTAGCCGTGGCGAG  GTTGGTGAGAGCATCAGCATGCGAACTAGCTCGGCAAGTCATGTGGATATTCAAGGAAAC  GAAGTAGTAAATCAATGGTACGCCGACATCAAGAATTACAACTTTGCTGAAGGCAAAGGTCCAGCGGGTAATTTCACTCAATTGGTCTGGAAAGCAACACGTGAGGTCGGATTCGGAAAG  GCACGGTCTTCTGGGAAATGTATTGTCGTTGCGCATTACCGACCACCGGGCAATGTTAGA  GGACACTATGCAGAGAATGTCGGTACCCCGACTGGCGAACAAGCGGCCTCGGTAGCCAGTGCTACGGACACAGGGAATCTGGACCCTAATGCTAAACGGACAGTGGTTACGGAAGAAGTCACCTCACCAGAAGGGAAGCGTTACACTGTTCACCGGGAGGTCATTGAAACCACAGAACCCGATGGTCATGTCAGAAGATGTGTCAACGAGACGTTCCAGGATTCACCCGATCAGGCCACTGCCGGTGGTAAACATGGCGCAAGTTCCGAAGCAGCCCACGGAGAAAATTTCGCTGATGCAGTGACCCGAGCGCACAACGTCTACCGGAAACGGCATGGTGTTGCGGATCTGCAATTAGACCCGGAGATTAGTCATATGGCGCAGGACTGGGCTGAACAGTTGGTAAATCGAGCCCACTTGTCGAACAGCGGGTTCACCTATCAAGGAGTACGTCTTGGAGAAAACGTCCTTTGTCGTTGGTCGAACACGGCTGCCACCGTTTCTGCCCAAGATGTCGTTGATCACTGGTATCAAGAAAGTTCCAAGTACAAGTTTAATTCGGAACCCAAAAGTATTCAAGGCATTGGCGGATTCACCCAGATGGTATGGAACGGATCACAACGCATCGGGGTTGGGATCGCTTCACAGGCAAAGAAGGATTTTTACAACCAGCCCAGCCAATCAAAAGTCATTGTTGTCTGCTTCTATTATCCACCCGGAAACGTCACCGGACAATTCAGAGCAAATGTGAAGCAAGGAATGAACTAG |
| C9 | ATGGTTTCCGACGAGTCGTGGGTTGTTCGTGAAAATATTTCCGATCCGGAGGCTTTGTTGCATATCGCGAAGTTGGCTGAACATGCCGAGCGCTTCACTGATATGGCAGCCGCTATGAAAAAGTTCACGGAACTGAAAAAACCGCTTAGCAACGATGAACGGAATCTTTTTTCCGTAGCT  TACAAAAATGTGGTGGGCGCTCGCCGGTCGGCCTGGCGTGTAATCACAAGCATCAAGAAC  AAGGATTCTGAGGATGAAAAGTCACCAACGAACGAACTTCGTCGAAAGATAGAGAACGAGCTTGAGCAAGTCTGCAAGGAGGTTTTGAACATCCTGGAGAAAAATTCTCTGCCATCTGAGACTACAGACGACGGACTGGTTTTTTATCACAAAATGAAAGGAGATTATTATCGTTATTTGGCTGAAGTACAAACGGGAGACAAGCGCAATGAATCCGTTCAGAAATCCCATCAGGCCTATGAAGAAGCCACGGAGAAAGCCAAACAATCTCTCAGTGAGACTCATCCTATTAGATTGGGACTAGCCCTAAACTACTCTGTATTCTACTACGAAATCGAGAATAATCCGGACAAGGCATGCGAATTGGCCAAGTCTGCCTTCGATAACGCCATAAGCCGGCTCGATCAAATCAAGGATGAGTCCTACAAAGACAGTACCTTGATTATGCAGCTACTTCGGGACAATTTGACGCTGTGGACGTCGGAGCGTGAAACTGACCAGTGA |
| C10 | ATGCTTCATTTCGCTCGCAATTCTTTACGGCTTTTGTCAGTGGCTCGTACACCTAATCTC  CGTTTCATCTCGGGGGCAGCACACACCCTTGAGGTCCGCCACAAAATCGAGGAAACTCGCGAAAAGATCGTACTTGGGGGTGGTCAGAAGCGTATCGATGCGCAGCACAAGCGTGGCAAGCTGACGGCGCGCGAACGTATCGACCTGTTGGCTGATCCTGACACATTTGTGGAGTACGACGCCTTCATGGAACATGATTGTCATGACTTTGACATGCAGAGACAGCGGGTCACGGGGGACAGTGTTGTTACCGGTCACTGCCAGGTCAATGGAAAAACTGTCTACCTATTCAGTCAAGACTTTACCGTATTTGGTGGAAGTTTGAGTCTGGTTCATGCTCGAAAGATCTGCAAGGTCTTG  GATCAAGCTATGCTCGTCGGAGCTCCTGTGATTGGTTTGAATGACTCTGGTGGTGCACGCATCCAAGAGGGCGTAGCGTCGCTGGCCGGCTATGCAGATATCTTCCAGCGGAACGTGAAC  GCCTCCGGTGTCATTCCACAAATCTCTCTCATCATGGGCCCCTGTGCTGGTGGTGCTGTT  TACTCCCCCGCTTTGACAGATTTTATTTTCATGGTTCAAGATACCTCATATATGTTCATCACTGGTCCAGATGTAGTCAAGTCGGTGACAAATGAGGACGTCACCCAAGATGAACTGGGT  GGTGCGAAGACTCATTGCAGTGTCTCTGGCGTAGCCCATAGAGCCTACCAGAACGACGTG  GAGGCCATACTCAGTGTTCGTGACTTCTTGACCTACTTGCCGTCGTCCAATAGGCAAAAATGCGCCCCACTGCGTGAATGTCACGATCCAGTGGACCGACTGGTTCCAACTTTAAATACG  ATTGTCCCACTGGAGCCTACCAGTGCCTACGATATGTACGAGGTCATCTATGCAATCGTG  GATGAGCGCGAGTTTTTCGAAATCATGCCTTCGTACGCGAAGAACCTCATCGTCGGATTCGCTCGCCTTGGAGGCCGCACAGTTGGTGTGGTTGCTAACCAACCCCGTGTATCAGCCGGC  TGCCTGGATATCAACTGTTCGGTGAAGGGTGCTCGATTCGTTCGTTTCTGTGATGCATTC  AACATCCCGCTCATCACATTTGTCGATGTTCCCGGTTTTCTGCCAGGAACTAGTCAGGAATACGGTGGGATTATTCGACATGGAGCGAAGCTTCTGTTCGCCTTTGCTGAGGCTACTGTG  CCTAAACTGACTGTGACCACGCGTAAATCGTACGGCGGAGCCTACTGTGTGATGAGTAGC  AAGCACCTCCGTGGGGATATCAATTATGCTTGGCCCTCAGCGGAGGTGGCTGTTATGGGTGCTAAGGGTGCGGTCCAAATCATTTTTCGTGGAAAAGAGGACCAGGCTCAGGCCGAAGAAGACTACATTCGAACATTTGCAAACCCATTCCCAGCTGCTGTTCGAGGTTATATGGATGAT  ATCCTAGATCCTCCACTTACACGGAAACGCCTTTGTCATGACCTGGAAATGCTGCAGACCAAGTCGCTGACGAACCCGTGGAAGAAACATGCCAATATGCCTTTGTAA |
| C11 | ATGGCGCAGCTAAATGCGATGTTCGGTCGCTTGGTCAAGTTGGGAGTTGGTATTGTTGCG  GCGGGAAGTATTCTGCCAATGGTGCTTTACAATGTTGATGGTGGGCATCGTGCCGTGATCTTTGATCGGTTCAAGGGCGTGCATCCTGAAGTTGTCGGTGAAGGGACACACTTCATCATA  CCTTGGGTACAGAAACCGATCATATTTGATATCCGCTCAAAGCCCCGAAACATTCCGGTT  ATGACGGGATCTAAAGATCTGCAGACTGTTAACATCACTCTGCGAATTCTCTTCCGGCCAGAGTCTTCATTGTTGCCCAAGATTTATCAGAACCTTGGTTTCGACTACGAGGAACGCGTT  CTACCGTCAATAACAACCGAAGTGCTCAAGGGTGTTGTAGCACAGTTTGATGCCAGTGAG  CTAATCACACAGCGAGAACTCGTGAGCCAGCGGGTTAATGATGATCTAACCGAACGTGCTTCTTCCTTTGGGATATTGCTGGACGATATTGCTTTAACACAAATCTCGTTCGGTCGAGAA  TTCTCTGAAGCCGTGGAAGCTAAACAAGTCGCACAACAAGAAGCCGAGCGCGCACGCTACCTAGTTGAAAAAGCAGAACAGCAAAAGCTGGCTGCTGTCATTACGGCTGGGGGCGATTCGGAAGCAGCTACACTTTTGGCCAAAGCATTCGGGTCGTCCGGGGAAGGTCTAATCGAGCTCAGACGAATCGAAGCAGCCGAGGATATTGCATATCAGCTGTCGAAAAACCGGAACGTTACTTACCTCCCCGAAGGCCAGCATACGCTACTCAATTTGCCTTCAGTTCAGACCTAA |
| C12 | ATGCCTACGGACAGAAAGTTCTTTGTTGGTGGAAACTGGAAAATGAATGGTAGTAAGAAG  GAAAACGATAAACTTATTGAAATGCTTACCCATGCCAAGATTGATCCAAACACAGAAGTTCTGGTTGCTCCACCGGCACTCTACCTCCCAAGCGTTCGTGAGAAGCTGGACAAACGCTTT  CATGTGGCCGCGCAAAACTGTTATAAGGTACCTTCCGGAGCTTTTACTGGTGAAGTAAGT  CCGGCTATGCTCAAAGATGTCGGCTGCGACTGGGTTATTCTTGGTCATTCAGAAAGACGC  CACATTCTGTTGGAGACAGACCAGTTGGTCGGCGAGAAGACAAATCATGCAATATCCGCCGGTGTTAATGTTATCGCATGCATAGGGGAGAAGTTGGAAGAACGAGAGGCGGGTAAAACTGAAGAGGTTTGCTTCAGACAAATGGAGGCCATACGAAAAAATCTGTCATCAGCTGACATGTGGAATCACATTGTCATCGCCTACGAACCTGTTTGGGCCATCGGAACAGGCAAGACGGCCACTGAGCAACAAGCGCAGGAGGTTCACCTGGCTGTTCGTAGATGGATGGAGGAGAAAGTCAGTCCAGCGGTCGCAAAAAGCATTCGGATCATATACGGAGGTTCAGTTACGGCTGCCAATTGCCGAACACTGGCCAAGCAACCAGATGTGGACGGCTTCCTCGTGGGTGGCGCATCGCTCAAACCGGATTTCATTGAAATTTGCAATGCGAATGCCTAG |
| C13 | ATGGACTCAGCTAAGAAACGGTGTCTCCGTACCCGTCGACCCAGTGATTCTTCCGAATCT  GAGGAGGAGAAAACCAATGATTCAAATGAGATTGTGCAGCAAGAGGACAGTACGCATGTAGTTGAAGCCATAAGGGAATTACAGAAGGTCCGTAAGCGACCTCCTGGTATCAGTCTTTCTGCTTTGAGTACTGGAAAAGCGGCTCCAGAAGAGACAATCATCGTTAGCGATCCCTTCAAGTTGAAAACGGGTGGATTAGTGGAGATTAGAAAAGCGATCAGGTCGAAGAAAACTGAAGAGGAGGATGATGTTGAAGCTCGTTTAGCTAAAACCTTCGCGACAGAGACTAACAAACGAGATGAAGATGCCGAAATGATTAAGTATATCGAAGAAGAAATCGCTCGACGGAAAGGATTACGACGTACTCCATCTCCAGAATCTAATGCTGGTGCAGACCTCCTTCGCGATGTGCCGGAATACTTAAGACCGGTTATCGGTCAACAAAAGGAGGATATGTTATCCAATCAAATGCTTTGTGGGATCCCCGAAGTTGACTTGGGTGTGGATGCGAAAATGCGCAACATTGAAGCAACCGAAGAGGCCAAGCAAACTTTGCTCAAGCATCGTTTCAACCGTGGATACGGAATGGCCTCTGATGGCCTGGCCCCAACTAATGTGGCCGTCAATTTCGTTCAGCACAGTCGATGGAATAGTCACAATGCGACGACAACCTTTTCATCTGGTGACTACACACGTGACCTTCTGAGTATTGCTTCGAAGGCCAATCCTCACAAGACGGACATTGTCCACCAGCAAACAACTGGACTTGATGCGGAACGCGAACGGTTGGGTGCCGAACGATCGACCGACAGTCTTGTATTACAGCGATTTAAGTCACACATGCGTGGGCGCAAGCGACGGTGA |
| C14 | ATGACGCAGTTTTTGCCGCCTAATCTGTTGGCGCTCTTCGCGCCCCGGGACCCGGTACCT  TTTCTCCCACCGATCGAAAAGCATGCGCACCATCGAAAATTACCCTATACCGGTGTTGCCCAATTTTTGGGTGAATTTGAAGACCCTTCAGAAACGAAACCAGCCGTTCGAATCGAAACA  CGAGAGGAACGGAAAGAGCGGAAGAGGAGAGAAAAACAAGAACAAGCAAATTACAAGCTCGAACAGGATCTTGCCCTATGGAATCCGAAAAAGAATCCTAGTGCCACATCCAACGCCTACAATACTATGTTTGTTGCCCGCATGAATTACGACACCAGTGAAGGAAAGCTACGTCGTGAGGCAGAAGCTTTCGGACGCGTTACCCAGATTGTGATGGTAAAAAACAGATTAACCGGTAAACCACGAGGATACGCATTCGTGGAGTTCGAGCATGAACGAGAGATGCACGCTGCTGTCAAAGGCCTAAACGGTAAAAAGATCGATGGAATGCGGATTCTAACCGACATTGAACGTGGAAGAACCCGACCGGATTGGAGGCCTCGACGTCTAGGTAAGGGTTTGGGTAAAAACCGTCAAGGGCCCAGTGAAAAACCCAAGGTCAGCAGCAAGCATGAGAACGGTCGTGAGCCTCCCAGTCACGGGCGTAGTTACGGACGTGCAACGGGATACATTCGAGAACGGGAGTTTGATCGCCGGAAACGGAGTCGTTCTCGTAGCCGTTCGCGCGACCGTGATCGACGACGGAGCCGCAGTCGGGACAGGTATCGAAAGCATCGTAGCCCTGAAGGTGATAGAAGCTTCAAACGTGGTCGTGACCGGCGTGAAGATATGATGCGACAGTATGGGGAGTATGGCGCGGAAATTCGAGCGGAGTACAGTGGAGATATGTGA |
